# Nailing femoral shaft fracture with postless distraction technique: a new technique enabled by shape-conforming pad

**DOI:** 10.1186/s10195-021-00573-z

**Published:** 2021-03-18

**Authors:** Alessandro Aprato, Davide Carlo Secco, Andrea D’Amelio, Elena Grosso, Alessandro Massè

**Affiliations:** University of Turin, Viale 25 aprile 137 int 6, 10133 Torino, Italy

**Keywords:** Femur fracture, Postless technique, Urological complications, Pudendal neurapraxia

## Abstract

**Background:**

Femoral shaft fractures are usually treated with nailing using a traction table and a perineal post, but this may occasionally result in various groin-related complications, including pudendal nerve neurapraxia. Although most of them are transient, complication rates of up to 26% are reported. Recently, postless distraction technique has been described for elective hip arthroscopy. In this study we compared post and postless distraction technique in femoral shaft fracture nailing in terms of (1) quality of reduction, (2) outcome, and (3) complications.

**Methods:**

We reviewed 50 patients treated with postless distraction nailing technique for femoral shaft fractures and compared them with our historical case series (95 patients). The following data were collected for all patients: age, gender, weight, height, diagnoses (fractures were classified according to the 2018 revision of AO classification), type and size of nail surgical timing, Trendelenburg angles during surgery, quality of reduction according to Baumgaertner and Thoresen classifications, Modified Harris Hip Scores at 6 months, and perineal complications.

**Results:**

Median age was 53 years, and median weight was 70 kg (range 50–103 kg). We found no significant difference in terms of quality of reduction (72 versus 74% “excellent” reduction for subtrochanteric fractures, while 81 versus 79% “excellent” reduction for femoral shaft fractures) and functional outcomes (Modified Harris Hip Score 74 versus 79). One patient in the control group had a failure of the fixation, and one patient in the postless group had a deep infection. Two patients in the control group reported pudendal nerve neurapraxia for 4 months, while none reported complication linked to the postless technique.

**Conclusions:**

Our results using the postless distraction technique show a sufficient distraction to allow reduction and internal fixation of the femoral fracture with a standard femoral nail.

**Level of evidence:**

IV

## Background

Femoral reduction and nailing for femoral shaft fractures is usually performed with a standard traction table using a perineal post. This technique may occasionally result in perineal complications, including pudendal nerve neurapraxia and vaginal or scrotal edema. Kao [1] and Rankin [2] reported respectively 15% and 27.6% of pudendal nerve palsy following femoral nailing. Those complications may be transient and recover within some months, or may end in permanent injury [3].

Postless distraction technique has already been described for hip arthroscopy and for associated femoral and pelvic fractures [4], and its safety and efficacy has been already described in three published papers [5, 6].

To the best of our knowledge, postless distraction systems have never been described in isolated femoral shaft fractures. The aim of this paper is to compare post and postless distraction technique in femoral shaft fracture nailing in terms of (1) quality of reduction, (2) outcome, and (3) complications.

## Methods

We reviewed 50 patients treated with postless distraction nailing technique for subtrochanteric and femoral shaft fractures and compared them with our historical case series (95 patients).

### Participants

The inclusion criteria were age > 18 years old and unilateral femoral shaft fracture without other fractures in the lower extremities. Patients with subtrochanteric (AO31A3) fractures were also included since they were candidates for the same surgical procedure (closed reduction and long nailing). The exclusion criteria were as follows: age > 65 years old, open fractures, pathological fractures, patients who did not consent to the use of postless technique, severe medical complications, and other associated severe injuries such as traumatic brain injury. A control group was selected using the same inclusion criteria as in our historical cohort, in which the patients were evaluated in a previous study to assess the outcome of our femoral nailing technique.

### Interventions

**Fig. 1:**
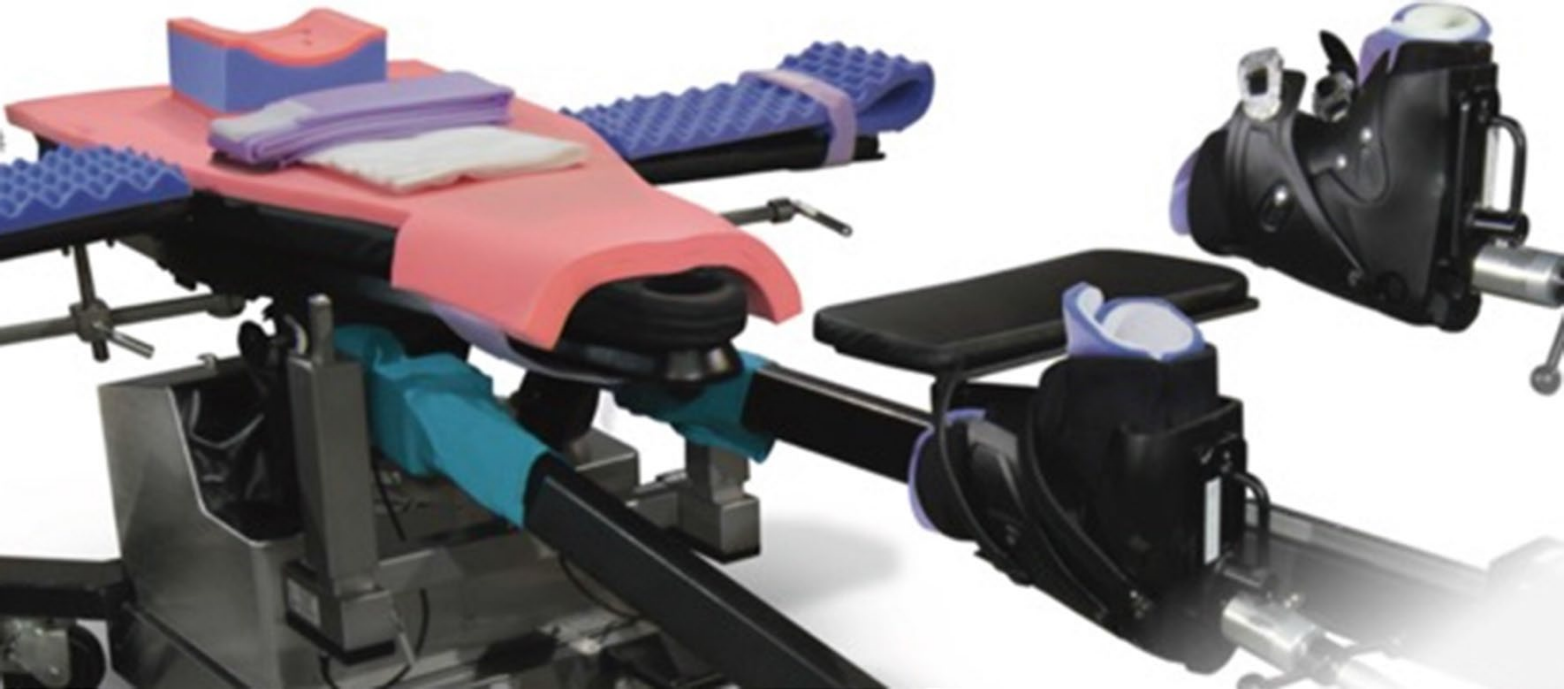
Shape-conforming hip positioning pad system was used to obtain an adequate distraction of the femoral fracture in the postless group

**Fig. 2:**
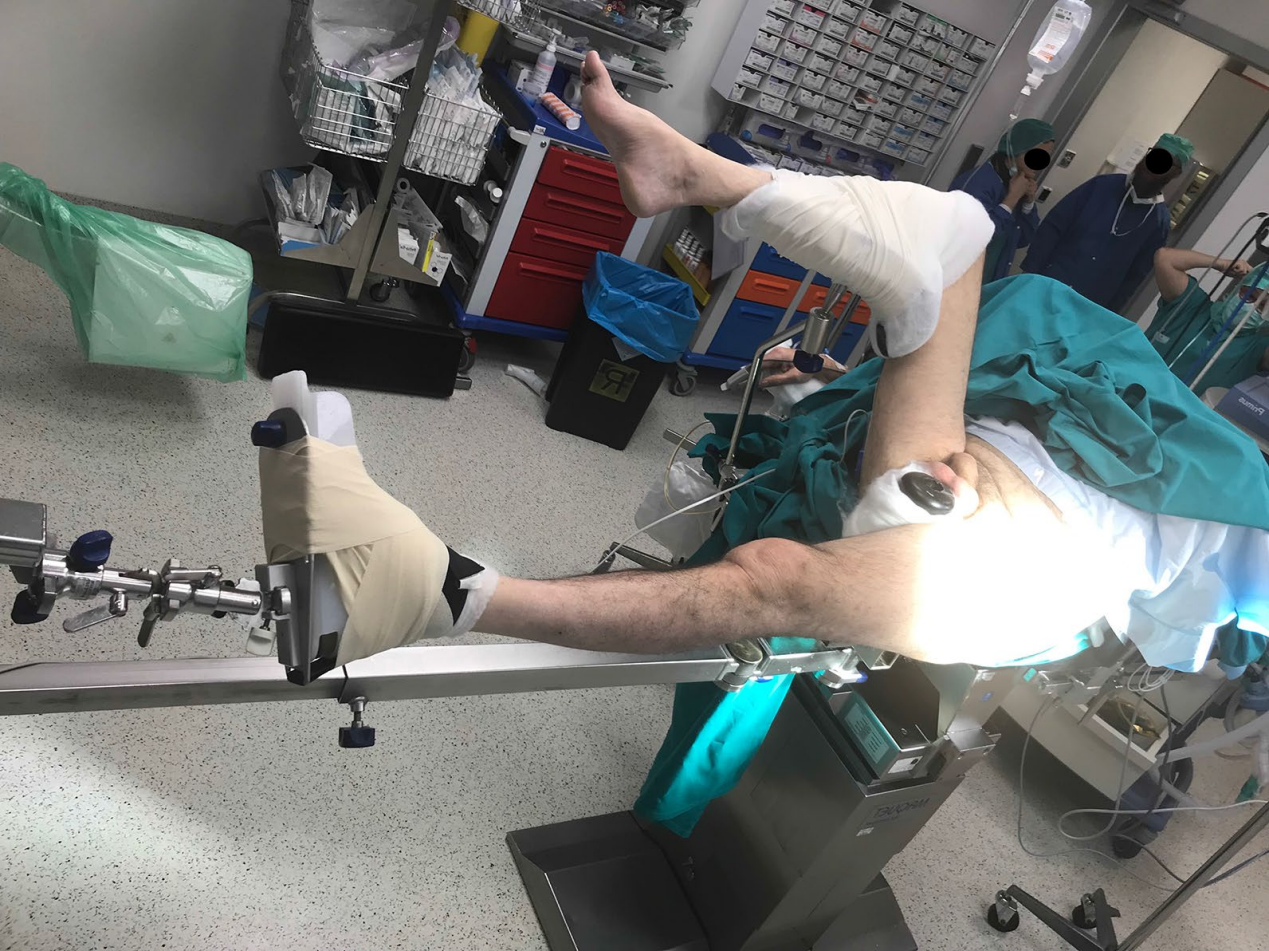
Patient’s positioning on the standard traction table with a perineal post

**Fig. 3:**
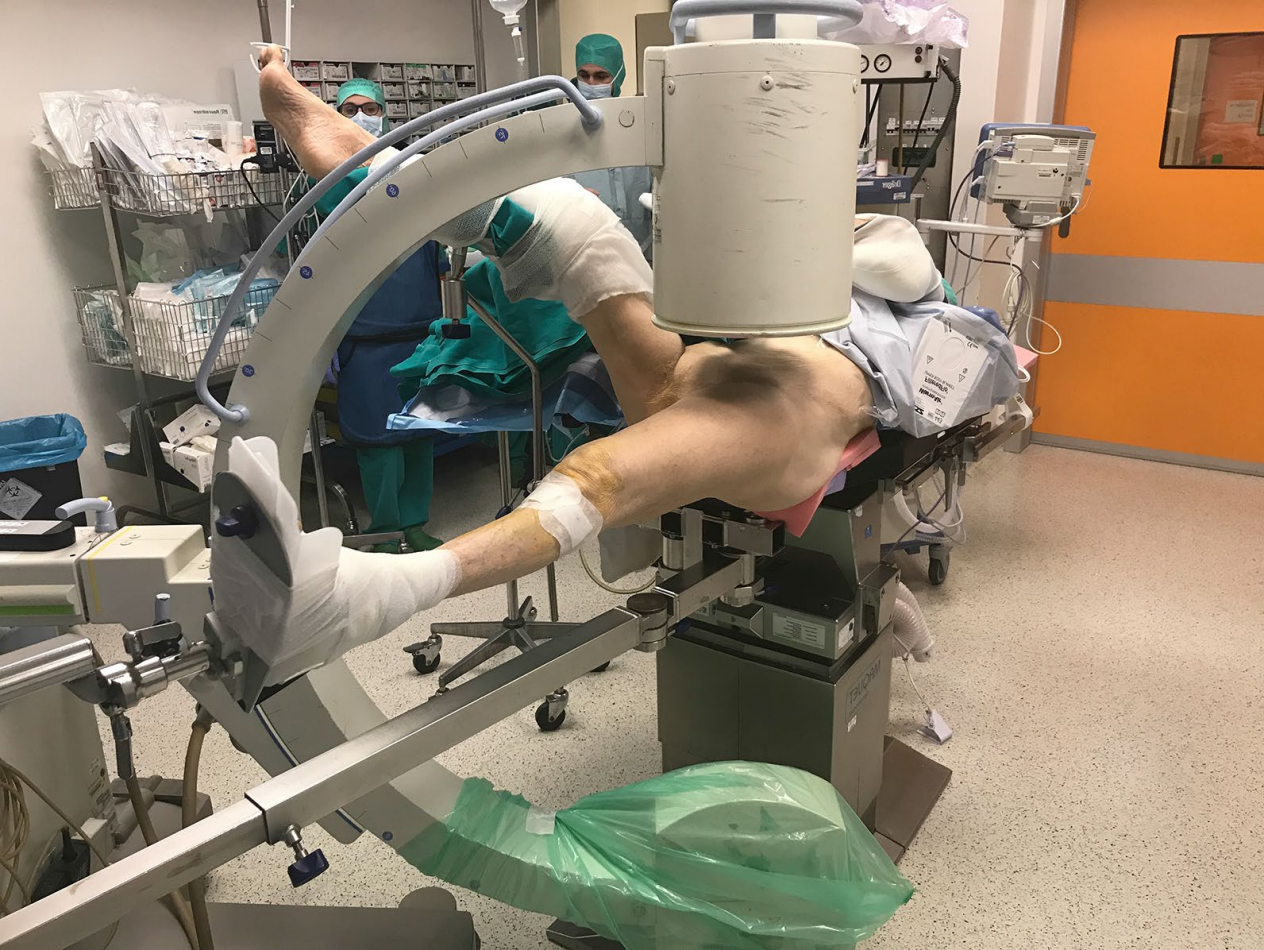
Patient’s positioning for the postless traction technique

**Fig. 4:**
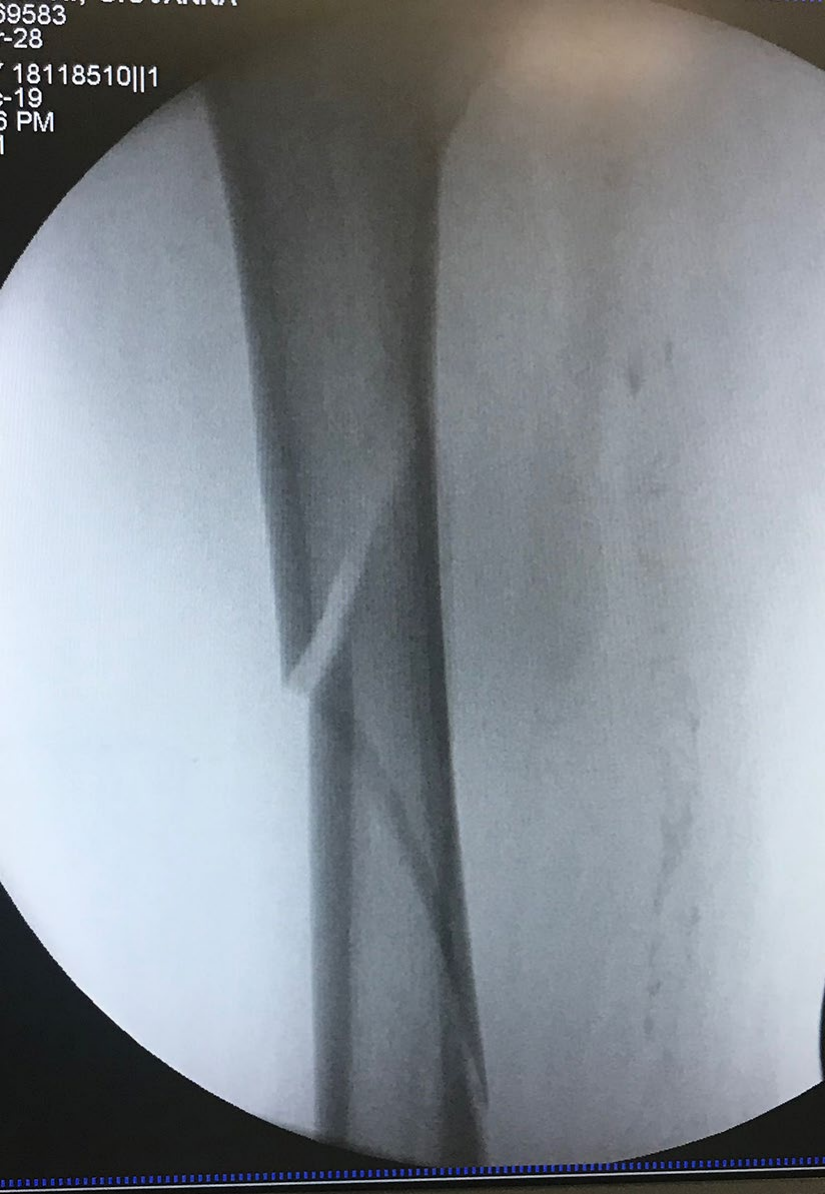
Intraoperative view of the obtained distraction in the postless group

A postless positioning system (Pink Hip Kit; Xodus Medical Inc., USA) with a shape-conforming hip positioning pad that prevents patient movement was used to achieve an adequate distraction of the femoral fracture in the postless group (Fig. 1), while the control group was treated with a standard traction table (NuovaBN traction Table, NUOVA BN S.r.l, Turin, Italy). The standard technique used in the control group has been described extensively in literature (Fig. 2). In the postless group, all patients were placed on a commercially available shape-conforming hip positioning pad and a standard traction system without the post was used (Fig. 3). The operative table was positioned at 5–10° of Trendelenburg based on the patient’s sex, height, weight, and fracture type. Trendelenburg position was used to increase the friction between patient and pad, allowing a stronger distraction. When positioning was completed, fractures were reduced according to their specific morphology with the most appropriate combination of traction, abduction, and rotation (Fig. 4). Femur fixations were performed with long femoral nails according to standard surgical technique [7]. All fractures were fixed with intramedullary nails (Trigen femoral nail; Smith&Nephew), subtrochanteric fractures were fixed proximally with two cephalic screws, and shaft fractures were fixed with a single trochanteric screw. Standard postoperative indications were suggested; immediate weight bearing was allowed.

### Outcomes

The following data were collected for all patients: age, gender, weight, diagnoses (fractures were classified according to the 2018 revision of AO classification) [8], type and size of nail, surgical timing, Trendelenburg angles during surgery, and quality of reduction according to Baumgaertner classification for subtrochanteric fracture [9, 10] and according to Thoresen classification for femoral shaft fracture [11]. Patients were interviewed at 6 months; the control group was evaluated as part of the outcome of a previous study, while the study group was interviewed to evaluate the outcome of the postless technique.

Modified Harris Hip Scores were determined telephonically, and patients were interviewed about perineal complications; they were asked postoperatively about the presence and duration of perineal numbness and, in males, erectile dysfunction.

### Statistical analysis

Categorical data were reported as frequencies and percentage, while continuous variables as mean and standard deviation if the distribution was normal or as median and interquartile range if the distribution was not normal. The normality of data was verified with the Shapiro–Wilk test. The two groups were compared with the *χ*^2^ test for categorical data. For continuous data, Student’s *t* test or Mann–Whitney *U* test were used, according to the distribution of the values. Statistical analysis was performed using StataMP13 (Stata Corp., College Station, TX.)

## Results

The historical control group was composed of 95 patients, while the postless group was composed of 50 patients. No patients were lost at follow-up.

Comparison of demographic data between the two groups is presented in Table 1. Mean waiting time from trauma to surgery was not significantly different between the two groups (38 h versus 41 h, *p* = 0.694).

**Table 1:** Baseline data

	Postless group	Control group	*p* value
Median age in years (IQ range)	54 years (range 40–62 years)	52 years (range 38–62 years)	0.211*
Median weight in kg (IQ range)	72 kg (range 61–95 kg)	78 kg (range 60–97 kg)	0.453*
Median BMI in kg/m^2^ (IQ range)	25.3 kg/m^2^ (range 20.7–29.2 kg/m^2^)	28.2 kg/m^2^ (range 22.1–31.0 kg/m^2^)	0.389*
Shaft/subtrochanteric fracture	29/21	42/53	0.429**
AO/OTA 32 A	8	20	
AO/OTA 32 B	8	18	
AO/OTA 32 C	5	15	
AO/OTA 31 A3	29	42	

^*^Mann–Whitney *U* test

^**^χ^2^ test

The mean surgical time for intramedullary nailing was 70 min and 73 min, respectively, in the postless and control group (*p* = 0.633). The mean Trendelenburg angle was 7° (range 0–11°) in the postless group. We did not report poor or insufficient distractions during femoral surgery in the postless group.

No significant differences were found in quality of reduction; subtrochanteric fracture reduction was defined as “excellent” in 21 (72%) and 31 (74%) patients, respectively, in study and control group (*p* = 0.502), while femoral shaft fracture reduction was classified as “excellent” in 17 (81%) and 41 (79%) patients, respectively, in study and control group (*p* = 0.087).

No significant differences were found in functional outcomes (Modified Harris Hip Score 96 versus 98,* p* = 0.438) between the two groups.

One patient in the control group had a failure of the fixation, and one patient in the postless group had a deep infection.

In the control group, 15 patients reported pudendal nerve neurapraxia with an average duration of 10 days, although only two of them reported a sensibility impairment of more than 2 months. Two patients (4% of the males) in the control group reported erectile dysfunction of 4 months. None reported pudendal complications in the postless group.

## Discussion

This study reports a technique for femur fracture reduction and nailing without a perineal post. Postless distraction technique has already been used in orthopedic elective surgery and by our team in combined pelvic and femoral fractures. Its safety and efficacy has already been described in hip arthroscopy and in combined femoral and pelvic fractures. In this case series, we report for the first time its use in femoral subtrochanteric and shaft fracture treatment. After our initial experience with this technique, we decided to use the device as routine in all patients with fractures who arrived at our hospital because we can always revert to a conventional traction table (add the post) if needed.

Although a traction table provides safe and appropriate patient positioning, it can also cause complications. In the past, manual reduction and traction without using a traction table has been advocated extensively to reduce those complications. Unfortunately, nailing in patients with unstable intertrochanteric fractures with manual traction method resulted in a significant increase in the number of surgical assistants required [12]; furthermore, the stable positioning and straightforward imaging with traction table resulted in a lower amount of radiation exposure and remained the most popular technique worldwide. Moreover, a lower rate of anatomic reduction has been demonstrated when a standard traction table was compared with the manual traction technique.

We think the postless technique is the optimal compromise between manual and standard table traction methods. It guarantees a stable and efficient traction without perineal complication. In our study, with this postless system, no patient developed new groin injuries or perineal neurological complications. Use of the postless distraction system may also prevent the development of pudendal nerve neurapraxia or erectile dysfunction.

Shape-conforming pad system did not hinder the correct positioning of the patient and allowed correct positioning of C-arm in either anteroposterior or lateral position.

Trendelenburg angles were moderate, did not negatively affect patients’ hemodynamics, and were not considered as dangerous by our anesthesiologists.

This postless system also some disadvantages. First of all, it is a single-use device, therefore increasing the cost of the procedure. Secondly, although not yet reported, there is a theoretical risk of patient’s fall, although this complication may be avoided if gentle traction is applied while checking the patient’s position continuously. Furthermore, the device must be tailored on the operating table and, at the moment, is not available for all the surgical tables. Moreover, the device does not protect the patient’s trunk from x-rays, as a leaded apron may not be placed under the patient body to protect it from radiation because otherwise the friction between the device and patient’s body would be strongly reduced.

There are some limitations in our study. Its main limitations are linked to its design. Functional evaluations were made by interview, no traction strength measure was recorded, and no evaluation of patients’ movement on the table (i.e., photographic analysis) was done.

## Conclusion

Distraction of femur fracture with standard perineal post may produce pudendal nerve injuries, while a postless system enables distraction, reduction, and nailing of femoral subtrochanteric or shaft fractures without pudendal nerve complications. This paper demonstrates the safety and efficacy of this technique.

## Data Availability

The datasets used and analyzed during the current study are available from the corresponding author on reasonable request.
